# Blood metabolites and gastric cancer risk: A bidirectional 2-sample Mendelian randomization study

**DOI:** 10.1097/MD.0000000000043339

**Published:** 2025-07-18

**Authors:** Sulan Chen, Bin Zhang, Song Wang, Ming Yang, Qiaohui Shen, Rui Zhang, Yan Leng

**Affiliations:** a College of Traditional Chinese Medicine, Changchun University of Chinese Medicine, Changchun, Jilin Province, China; b Department of Hepatosplenogastrology, The Affiliated Hospital of Changchun University of Chinese Medicine, Changchun, Jilin Province, China.

**Keywords:** biomarkers, blood metabolites, gastric cancer, Mendelian randomization

## Abstract

Notably, metabolic dysregulation stands as a prominent characteristic of cancer. The identification of biomarkers through blood metabolomics presents a novel approach for the diagnosis and treatment of gastric cancer. We performed a 2-sample Mendelian randomization (MR) analysis to assess the causality from genetically proxied 486 blood metabolites to gastric cancer. In this study, MR analysis was employed to assess the correlation between 486 serum metabolites and gastric cancer. Five different methods, namely inverse-variance weighting, MR-Egger method, weighted median method, simple mode method, and weighted mode method, were utilized for evaluation. Sensitivity analysis was conducted, encompassing heterogeneity testing, horizontal pleiotropy testing, and leave-one-out testing. Furthermore, the study encompassed linkage disequilibrium score (LDSC) genetic association and directionality assessment, metabolic pathway analysis, and reverse MR analysis. The findings revealed the presence of 21 metabolites, comprising 14 known metabolites and 7 unidentified metabolites that potentially play a causal role in gastric cancer. The reverse MR and directional assessment indicated the absence of reverse causality between gastric cancer and the candidate metabolites. Moreover, the LDSC genetic association solely identified a genetic association between gastric cancer and the unknown metabolite X-11315. Additionally, the metabolic pathway analysis identified 3 pathways that may be implicated in the development of the disease. We observed negative correlations between 12 serum metabolites and the risk of gastric cancer, while 9 serum metabolites showed positive correlations. Notably, 3-methyl-2-oxovalerate exhibited promising therapeutic potential, whereas 2-aminobutyrate displayed a higher risk factor. The integration of genomics and metabolomics in our investigation offers novel insights into the underlying mechanisms of gastric cancer, thereby holding significant implications for the screening and prevention of this disease.

## 1. Introduction

Gastric cancer represents a pressing global health concern. With the latest estimate from GLOBOCAN projecting a total of 1.089 million cases worldwide by 2020. Gastric cancer remains the fifth most prevalent cause of cancer-related mortality.^[1]^ Consequently, comprehending the pathogenesis of gastric cancer, discerning potential biomarkers for early detection, and uncovering risk factors for prevention and treatment are of paramount importance in addressing the prevention and management of gastric cancer.

While chronic Helicobacter pylori infection serves as the primary etiological factor for gastric cancer, excessive alcohol consumption, smoking, obesity, type 2 diabetes, and metabolic dysfunction have also been identified as factors that heighten the risk of gastric cancer, all of which are associated with metabolic disorders.^[1–4]^ The emergence of a distinctive metabolic profile in cancer cells has prompted the utilization of metabolomics as a prompt and efficient approach for the detection of new cancer biomarkers, thereby offering insights into the prediction of cancer susceptibility and underlying molecular mechanisms^.[5,6]^ Notably, alterations in blood metabolites play a pivotal role in cancer progression, with mounting evidence suggesting that blood metabolomics analysis holds promise in guiding the diagnosis, staging, treatment selection, and prognosis assessment for individuals with gastric cancer.^[7]^ Research has indicated that gastric cancer is distinguished by dysregulation in glucose metabolism, amino acid metabolism, lipid metabolism, nucleotide metabolism, and other metabolic irregularities.^[8]^ In individuals with gastric cancer, there is an upregulation of glucose transporters, an enhancement in glucose metabolism, and a decrease in glucose content compared to healthy controls.^[9]^ Furthermore, amino acids serve as alternative energy sources for the proliferation of cancer cells, and investigations have demonstrated that aberrant metabolism of amino acids such as tryptophan, alanine, glutamine, arginine, and serine frequently manifest in gastric cancer.^[10–13]^ Fatty acid synthase is found to be overexpressed in gastric cancer. In comparison to benign gastric tissues, there is an observed increase in the levels of fatty acids such as octadecanoic acid, docosahexaenoic acid, and β-hydroxybutyric acid.^[14]^ Nucleotide metabolism is identified as the final and crucial factor in the replication of malignant cells. Liu et al discovered that cytidine, inosine, uric acid, and xanthine were down-regulated in gastric cancer samples, while N6-methyladenosine, and uracil were up-regulated in gastric cancer samples.^[15]^ Furthermore, the microbial composition of the gastric environment may generate metabolites that exert an impact on the metabolic processes of cancer cells.^[16]^ Nevertheless, the presence of unidentified confounding variables and the potential influence of reverse causality introduce uncertainty regarding the existence of a definitive correlation between the diverse metabolite findings derived from observational studies and gastric cancer, as well as the causality of this relationship.

Shin et al conducted an extensive genome-wide association studies (GWAS) on human metabolites, which stands as the most comprehensive study of its kind to date.^[17]^ The utilization of the summary statistics derived from this GWAS has enabled numerous researchers to establish the causal relationship between a diverse range of blood metabolites and various diseases, encompassing autoimmune diseases, neuroticism, lacunar stroke, epilepsy, osteoporosis, colorectal cancer, among others.^[18–23]^ Two-sample Mendelian randomization (MR) analysis was employed in this study to investigate the causal relationship between blood metabolites and gastric cancer (gastric cancer), as well as to identify blood metabolites with a causal association with gastric cancer. The findings of this study can serve as a reference for early screening and treatment of gastric cancer.

## 2. Materials and methods

### 2.1. Study framework and overall design

**Figure 1.**
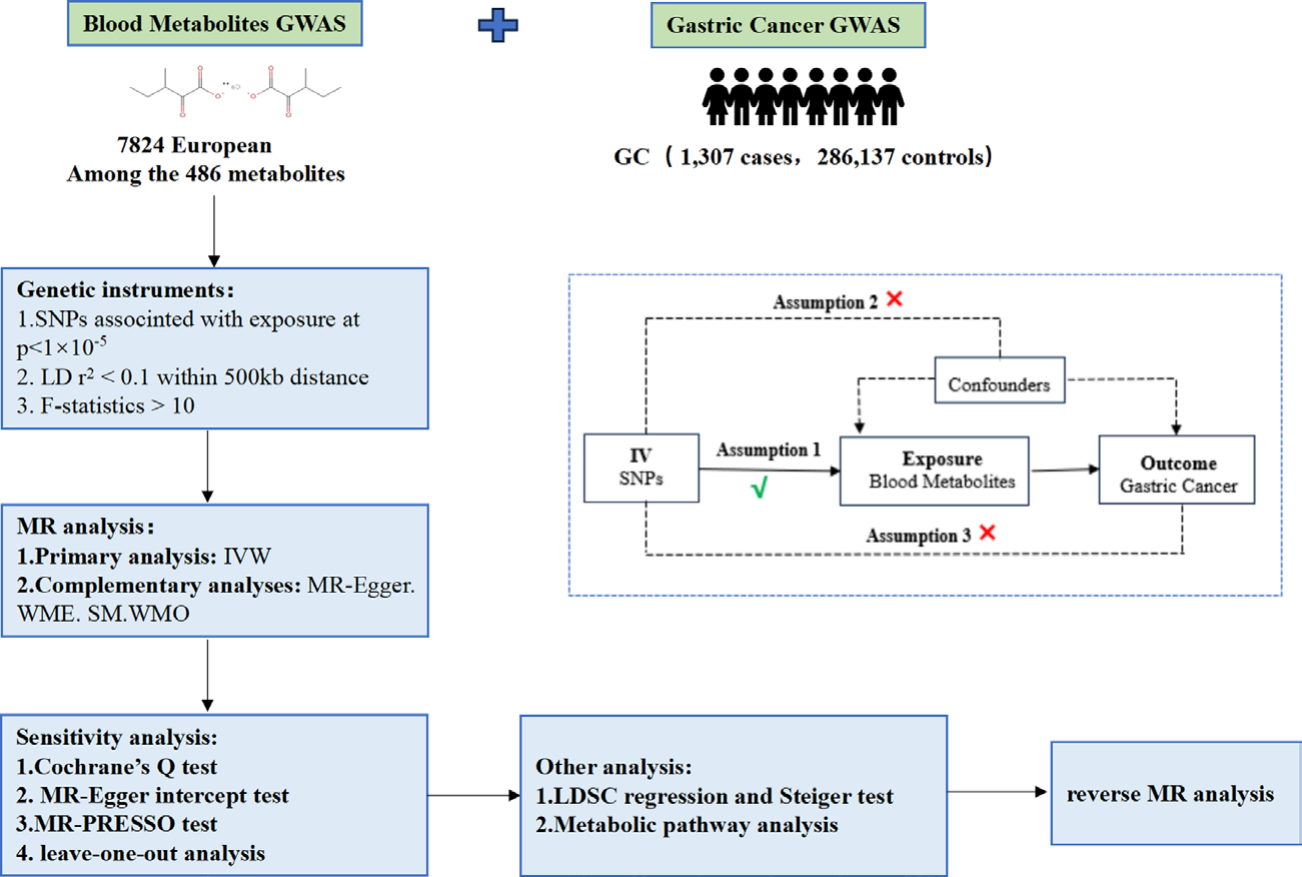
Overview of present MR analyses and assumptions. GWAS = genome-wide association studies, IV = instrumental variable, IVW = inverse-variance weighting, LDSC = linkage disequilibrium score, MR = Mendelian randomization, SM = simple mode method, SNPs = single nucleotide polymorphisms, WME = weighted median method, WMO = weighted mode method.

In this investigation, a 2-sample MR Analysis approach was employed to assess the plausible causal association between 486 metabolites and gastric cancer in blood. The GWAS were publicly accessible, this study utilized the GWAS dataset for secondary analyses, thereby obviating the need for additional ethical approval. The schematic representation of the overall study design is depicted in Figure 1.

### 2.2. Sources and descriptions of GWAS datasets

The GWAS data pertaining to human blood metabolites were derived from the most comprehensive investigation conducted by Shin et al^[17]^ to date, which extensively explored the genetic loci that significantly influence human metabolism. The study utilized data from the KORA and TwinsUK cohorts, comprising a sample size of 7824 European adults. Tandem mass spectrometry was employed to analyze a comprehensive set of 2.1 million single nucleotide polymorphisms (SNPs). Additionally, 486 blood metabolites (consisting of 177 unknown metabolites and 309 known metabolites) were successfully acquired and subsequently categorized into 8 distinct metabolic groups, namely nucleotide, amino acid, carbohydrate, vitamin, lipid, energy, peptide, cofactor, and xenobiotic metabolism. The GWAS statistics for gastric cancer were obtained from the most recent version R9 of the FinnGen database, encompassing a total sample size of 288,444 individuals of European descent. This sample included 1370 cases and 286,137 controls.

### 2.3. Instrumental variables screening and selection assessment

Three key assumptions for IVs selection: in this study, the IVs need to satisfy the following 3 key assumptions: strong correlation between IVs and blood metabolites: we selected SNPs that were strongly associated with blood metabolites. Initially, we used a strict threshold of *P* < 5 × 10^−8^ for SNPs selection. However, no SNPs met this stringent criterion, so we relaxed the threshold to *P* < 1 × 10^−5^ to include potential IVs that were sufficiently correlated with the metabolites of interest.^[24]^ IVs are independent of each other and confounders: the selection of IVs cannot be associated with other factors that influence the relationship between blood metabolites and gastric cancer. We ensured that the selected SNPs were independent of each other and confounders, and for this purpose we excluded SNPs with strong linkage disequilibrium with other SNPs, set the parameter of LD to *r*^2^ < 0.01, and restricted the genetic distances to within 500 kb. IVs should affect gastric cancer risk only through blood metabolites, not through other pathways: we ensured that the selected SNPs influenced gastric cancer risk only via blood metabolites by aligning the effect alleles between the exposure (blood metabolites) and the outcome (gastric cancer) datasets. Additionally, we removed any duplicate SNPs to avoid the potential for spurious associations. Furthermore, the *F*-statistic was computed for each SNPs, with a threshold of *F*-statistic > 10 being employed to mitigate any potential bias stemming from inadequate instruments.^[25]^ This process ensures that the IVs used in our analysis are robust and can provide reliable causal inferences.

### 2.4. Five methods of Mendelian randomization analysis

To investigate the potential causal association between blood metabolites and gastric cancer, this study employed 5 complementary approaches, namely inverse-variance weighting (IVW), MR-Egger method, weighted median method, simple mode method, and weighted mode method. Among these methods, the IVW approach demonstrated superior accuracy and effectiveness compared to the remaining 4 methods. Hence, the initial preference was given to the IVW method, while the outcomes of alternative methods were utilized as supplementary information. The IVW approach aggregates the effect estimates of all valid SNPs, ensuring unbiased results in the absence of horizontal pleiotropy.^[26]^ However, the MR-Egger method addresses the limitations of the IVW method by accommodating pleiotropy, albeit with a potential increase in the biased frontal type 1 error rate.^[27]^ Moreover, when the effective instrumental variables contribute to more than 50% of the weight of weighted median method, a consistent estimation of causal effects can be achieved.^[28]^ The methods of simple mode method, and weighted mode method are utilized in clustering SNPs with comparable causal effects and estimating the causal effects of the majority of clustered SNPs.^[29]^

### 2.5. Multiple sensitivity analysis and metabolite efficacy level calculation

Multiple sensitivity analysis techniques were employed to ascertain the potential presence of pleiotropy. Initially, the presence of pleiotropy in the IV was evaluated using the MR-Egger intercept method. If the intercept approached 0 (<0.1) and the *P*-value was <.05, it was concluded that there was no substantial evidence of horizontal pleiotropy in the test. Furthermore, MR-PRESSO was employed to identify outliers and assess horizontal pleiotropy.^[30]^ The heterogeneity of SNPs was assessed through Cochran *Q* test. *P* > .05 suggests the absence of heterogeneity, leading to the utilization of the fixed effect model. Conversely, *P* < .05 indicates the presence of heterogeneity, necessitating the adoption of the random effect model.^[31]^ Following the heterogeneity and horizontal pleiotropy tests, a leave-one-out method was employed for sensitivity analysis. This approach aimed to investigate the impact of individual SNPs on metabolites and assess the reliability and consistency of the findings.^[32]^ Furthermore, upon computing the odds ratio for each candidate metabolite, it was observed that certain metabolites exhibited excessively high odds ratio (OR) values. To address this, we employed the MR effect level (Power) website (https://shiny.cnsgenomics.com/mRnd) to assess the efficacy of each metabolite level, thereby enhancing the ability to detect genuine effects. Specifically, if the effect level exceeded 0.8, the study design was deemed to possess an 80% likelihood of detecting a true effect.^[33]^

### 2.6. Genetic association analysis and causality direction assessment

The LDSC regression method, widely employed in genetic association studies, is utilized to assess the genetic associations and determine the directionality. This method enables the estimation and adjustment for confounding factors, thereby distinguishing genuine polygenic effects from confounding biases, including hidden associations and population stratification.^[34,35]^ In our analysis, we examined the impact of the IVW approach on the genetic association between candidate metabolism and gastric cancer, specifically focusing on those associations that met the sensitivity analysis criteria and had a significance level of *P* < .05. Furthermore, to mitigate the potential bias arising from reverse causality, an additional Steiger test was conducted.

### 2.7. Metabolic pathway analysis methods and databases

The pathogenesis of gastric cancer was explored using MetaboAnalyst5.0 metabolic pathway analysis, which encompassed the small molecule pathway database and KEGG database.^[36–38]^

### 2.8. Reverse Mendelian randomization analysis

In order to examine the presence of reverse causality between the identified blood metabolites and gastric cancer, a reverse MR analysis was performed, with gastric cancer as the exposure and the candidate metabolites identified in the preceding stage as the outcome.

## 3. Results

### 3.1. MR analysis

**Figure 2.**
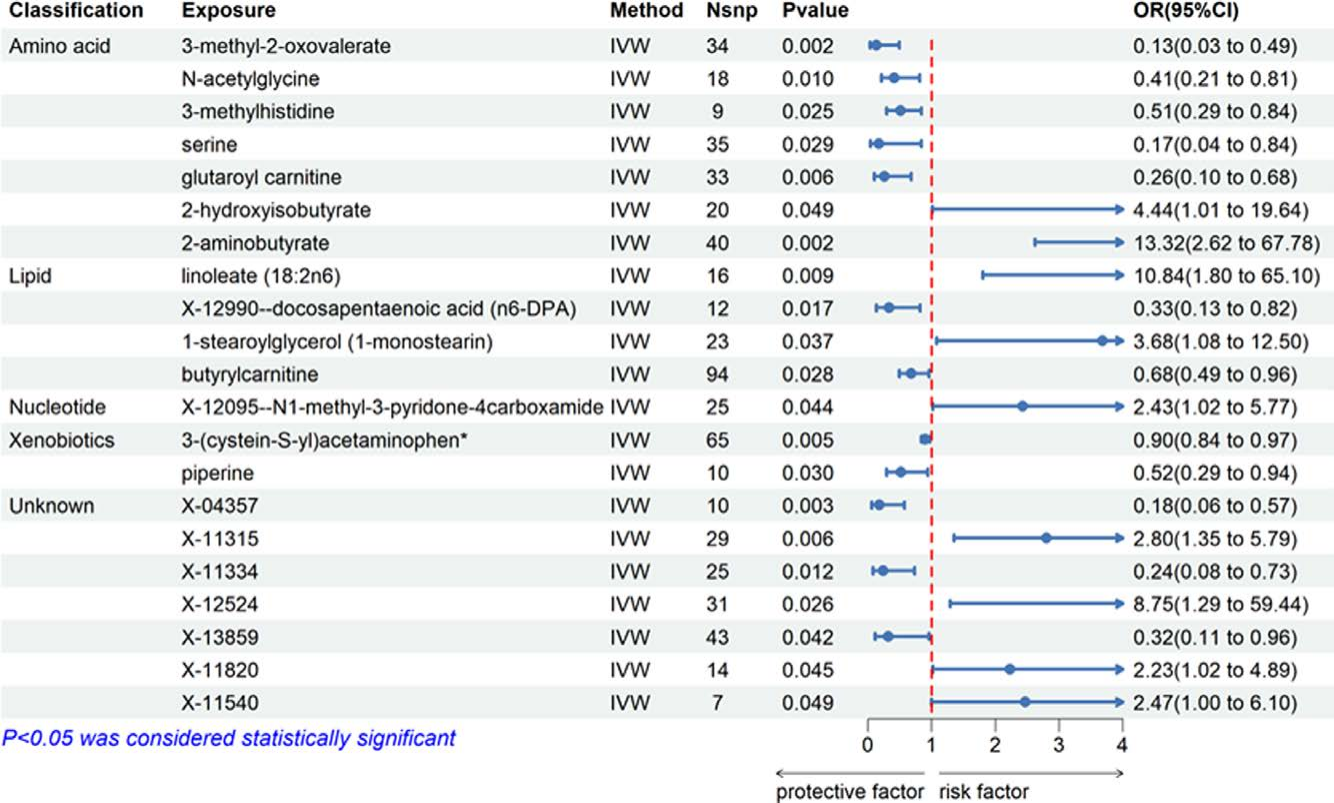
Mendelian randomization results for blood metabolites and the risks of gastric cancer based on the IVW method. The dashed red and solid blue lines indicate MR ORs and 95% CIs. The blue dots represent MR. CI = confidence interval, IVW = inverse-variance weighting, MR = Mendelian randomization, OR = odds ratio.

According to the results obtained from the IVW analysis, a total of 21 metabolites were found to have a significant association with gastric cancer (*P* < .05). These metabolites comprised of 7 amino acids, 4 lipids, 1 nucleotide, 2 exogenous metabolites, and 7 unknown metabolites. The *F*-statistics for all SNPs used as instrumental variables exceeded the empirical threshold of 10, indicating their strong instrumental nature. However, these associations did not meet the criteria for significance after applying the conservative Bonferroni correction. Consequently, a more lenient false discovery rate correction was employed, revealing that all 21 metabolites remained statistically significant. Finally, we have identified a total of 21 metabolites that have met the rigorous screening criteria and can be considered as potential candidate metabolites (Fig. 2 and Table S1, Supplemental Digital Content, https://links.lww.com/MD/P399).

### 3.2. Sensitivity analysis

To ensure the validity of our findings, we conducted multiple sensitivity analyses, including the MR-Egger intercept analysis on a pool of 21 candidate blood metabolites. The MR-Egger intercept was found to be close to 0 (<0.1), suggesting the absence of horizontal pleiotropy. However, the presence of an outlier value in 2-aminobutyrate was detected using the MR-PRESSO method. Consequently, we removed the SNP rs4717210 and repeated the MR analysis. The subsequent MR-PRESSO analysis revealed *P* > .05, indicating the absence of horizontal pleiotropy after removing the outlier SNPs. The Cochran *Q* test indicated no evidence of heterogeneity (all *P* > .05). The leave-one-out method had minimal impact on the results when SNPs were sequentially removed, suggesting the robustness of the findings. Additionally, the Power value exceeding 0.8 suggests that the study design is capable of effectively detecting the true effect (Figs. S1 and S2, Supplemental Digital Content, https://links.lww.com/MD/P401, Table S2, Supplemental Digital Content, https://links.lww.com/MD/P399).

### 3.3. LDSC and directionality assessment

Regarding the LDSC results, we observed a correlation solely between X-11315, an unidentified metabolite, and gastric cancer (rg = −1.691, rg_se = 0.771, rg_*p* = 0.028) (Table S3, Supplemental Digital Content, https://links.lww.com/MD/P399). These LDSC findings provide weak evidence for a genetic factor. However, it is important to acknowledge that the small sample size of metabolites may introduce a potential limitation in terms of statistical power. To address this concern, we conducted the Steiger test to validate the direction of action of the metabolites on gastric cancer. The Steiger test showed that all 21 metabolites had directionality marked as TRUE, indicating that the instrumental variables explained more variance in the exposure (metabolites) than in the outcome (gastric cancer), supporting a causal direction from metabolite to disease. Most *P*-values were <.01. The *P*-value for butyrylcarnitine is 0, which may be due to it being highly significant and rounded during the calculation. The *P*-value for 3-(cystein-S-yl) acetaminophen is NA, which may be due to missing data or insufficient explained variance. Nevertheless, its directionality is still marked as TRUE, indicating no evidence of reverse causality. For the remaining metabolites, the *P*-values are all <.01.

### 3.4. Metabolic pathway analysis

Our study successfully identifies 3 significant metabolic pathways associated with the pathogenesis of gastric cancer, Linoleate was found to be significantly associated with the metabolism of linoleic acid (*P* = .01). Similarly, 3-methyl-2-oxovalerate was found to be involved in the metabolic pathway of valine, leucine, and isoleucine (*P* = .02). Additionally, 3-methylhistidine was found to be involved in histidine metabolism (*P* = .04) (Table S4, Supplemental Digital Content, https://links.lww.com/MD/P399).

### 3.5. Reverse MR Analysis

To further rule out the possibility of reverse causality, we conducted a reverse MR analysis by treating gastric cancer as the exposure and the 21 candidate blood metabolites as the outcomes. The IVW method was used to estimate the reverse causal effects. The results showed that all IVW methods had *P*-values >.05, indicating that gastric cancer does not have a significant reverse causal effect on the levels of these blood metabolites. (Table S5, Supplemental Digital Content, https://links.lww.com/MD/P399).

## 4. Discussion

In this study, we conducted an integration of the summary statistics obtained from 2 extensive GWAS focusing on blood metabolites and gastric cancer. Our objective was to systematically elucidate the genetic perspective underlying the potential mechanism of association between blood metabolites and gastric cancer. Employing genetic variants as IVs, we successfully identified 21 metabolites that exhibit potential causal relationships with gastric cancer using the MR method. Among these metabolites, 14 are already known (comprising 7 amino acids, 4 lipids, 1 nucleotide, and 2 exogenous metabolites), while the remaining 7 metabolites are yet to be characterized. 3-methyl-2-oxovalerate exhibits significance across 5 MR Methods and can be considered a prominent metabolite with a crucial association with gastric cancer.

3-Methyl-2-oxovalerate is a branched-chain amino acids (BCAAs) metabolic intermediate involved in the catabolism of leucine, valine and isoleucine.^[39]^ Its anticancer effects may have potential anticancer properties by affecting cellular metabolism through inhibition of ketoglutarate dehydrogenase complex.^[40]^ In the present study, 3-methyl-2-oxovalerate was observed to be negatively correlated with the risk of gastric cancer with potential protective effects. This is consistent with studies suggesting that BCAAs may have anticancer properties by improving cellular metabolism and reducing oxidative stress. Studies have shown that BCAAs supplementation helps to improve muscle atrophy in cancer patients, including those with gastric cancer.^[41]^ Our findings provide further support for the potential therapeutic role of 3-methyl-2-oxovalerate in cancer treatment. Regrettably, the contribution of 3-methyl-2-oxovalerate to the pathophysiological mechanisms of gastric cancer remains significantly constrained. The identification of 3-methyl-2-oxovalerate would be a highly informative breakthrough, warranting further exploration into its protective mechanism against gastric cancer.

Serine is an amino acid involved in 1-carbon metabolism and is crucial for nucleotide and protein synthesis. In normal cells, serine can be synthesized through glucose or glycine pathways, but in the tumor microenvironment, cancer cells are highly dependent on exogenous serine uptake due to their high proliferation demands.^[42]^ Limiting dietary serine leads to the activation of endogenous serine synthesis pathways, which can inhibit tumor growth through 2 mechanisms: first, activation of the glucose pathway for serine synthesis diverts intermediates of glycolysis, such as 3-phosphoglycerate, reducing energy supply^[43]^; second, the glycine pathway forces serine hydroxymethyltransferase to catalyze the reaction in reverse, hindering nucleotide synthesis and inducing 1-deoxysphingolipid accumulation, thus inhibiting proliferation.^[44–46]^ Studies have found that high concentrations of serine (150 mg/mL) can directly promote invasion and lymph node metastasis in gastric cancer cells,^[47]^ suggesting a potential cancer-promoting risk. However, it is noteworthy that in studies on cisplatin-resistant gastric cancer, the addition of serine was beneficial. This is related to the role of phosphoglycerate dehydrogenase (PHGDH), a key enzyme in serine metabolism. PHGDH is highly expressed in various cancers and promotes tumor progression, and its inhibitors can suppress the growth of multiple cancers, increasing sensitivity to chemotherapy drugs.^[48–57]^ However, reducing serine levels or inhibiting PHGDH hinders the toxicity and pro-apoptotic effects of cisplatin on gastric cancer cells.^[58]^ The mechanisms by which serine metabolism affects chemotherapy sensitivity in gastric cancer may differ from other cancers. Research suggests that the mechanism may involve the suppression of serine metabolism, which lowers H3K4 trimethylation and increases chromatin density, thereby reducing the toxicity and pro-apoptotic effects of platinum-based chemotherapy drugs on gastric cancer cells.^[59]^ Overall, the activation of endogenous serine synthesis pathways inhibits the proliferation ability and rate of cancer cells, thus exerting an anticancer effect. In studies of cisplatin resistance, the addition of serine alleviates resistance in gastric cancer patients, which is beneficial for their treatment. This is consistent with our study, which suggests that serine is a protective factor in gastric cancer. However, under other conditions, especially at high concentrations, serine may promote tumor growth and spread. Further in-depth studies are needed to evaluate the potential impact of exogenous serine supplementation on gastric cancer treatment.

Piperine, a natural alkaloid extracted from chili peppers, exhibits significant antioxidant, anti-inflammatory, and antitumor properties. Studies indicate that piperine inhibits tumor cell growth through multiple mechanisms and demonstrates anticancer effects across various cancer types.^[60–65]^ Its protective role in gastric cancer has also been confirmed by multi-mechanistic investigations. Experimental evidence reveals that piperine suppresses gastric cancer cell proliferation and induces apoptosis by inhibiting the PI3K/Akt signaling pathway,^[66]^ while additional studies demonstrate its ability to downregulate IL-6 expression and inhibit TMK-1 cell invasion via blockade of the p38/MAPK/STAT3 pathway.^[67]^ Furthermore, piperine induces dose-dependent apoptosis in H-27 gastric cancer cells through reactive oxygen species accumulation and mitochondrial damage.^[68]^ Our study similarly identifies piperine as a protective factor against gastric cancer, showing a negative correlation with gastric cancer risk. Notably, piperine exerts dual inhibitory effects against Helicobacter pylori, a major gastric carcinogen: it completely suppresses bacterial growth at 125 to 250 μM concentrations and disrupts bacterial adhesion to host cells by targeting virulence-associated molecules such as AlpA-B and BabA.^[69]^ Additionally, piperine enhances the efficacy of various chemotherapeutic agents and other natural compounds with anticancer properties.^[70–75]^ Research also indicates that piperine exerts specific cytotoxic effects on cancer cells without affecting normal cells and may restore senescent cells adjacent to malignancies.^[76]^ However, its clinical translation is limited by hydrophobicity and low bioavailability.^[77]^ Current advancements in nanoformulations (e.g., nanoemulsions and nanocapsules) have partially improved delivery efficiency and mitigated toxicity concerns,^[78–81]^ yet further clinical studies are required to validate its safety profile and therapeutic potential.

2-Hydroxyisobutyrate is a nonessential amino acid involved in nitrogen metabolism and energy production, primarily derived from branched-chain amino acid metabolism and fatty acid oxidation. It serves as a precursor for the synthesis of acetyl-CoA. Under normal physiological conditions, its concentration remains low; however, it may significantly accumulate in metabolic abnormalities such as the tumor microenvironment.^[82]^ Chan et al conducted a comprehensive analysis of urine samples obtained from individuals diagnosed with gastric cancer, those with benign gastric lesions, and healthy individuals. Through the integration of 3 metabolites, namely 2-hydroxyisobutyrate, 3-indole sulfate, and alanine, the researchers successfully devised a reliable method for distinguishing between gastric cancer patients and healthy individuals. Notably, the area under the receiver operating characteristic curve reached an impressive value of 0.95.^[83]^ In line with the findings of this study, our research demonstrated a positive correlation between 2-hydroxyisobutyric acid and increased vulnerability to gastric cancer, suggesting its potential utility as a diagnostic biomarker for gastric cancer in clinical practice.

In our study, we found that linoleic acid is a risk factor for gastric cancer, with a positive correlation to gastric cancer risk. Matsuoka et al^[84]^ observed that in a gastric cancer mouse model, the incidence of peritoneal metastasis of gastric cancer cells significantly increased in a dose-dependent manner as the dietary linoleic acid content increased (6%, 8%, 10%, 12%). This effect was mediated by COX-2-mediated arachidonic acid metabolism, promoting ERK phosphorylation and the expression of matrix metalloproteinase-2, thus enhancing tumor invasiveness. Additionally, Edin et al^[85]^ also found that high doses (12%) of linoleic acid in a gastric cancer mouse model enhanced tumor angiogenesis and invasiveness, especially through the PAI-1 pathway, by promoting inflammation. However, in cellular studies, the effects of linoleic acid on gastric cancer cells were different. Choi et al^[86]^ found that at low concentrations (50–200 µM), linoleic acid significantly inhibited the growth of AGS human gastric adenocarcinoma cells by downregulating COX-2 expression, reducing PGE_2_ synthesis, and suppressing telomerase activity, leading to apoptosis. Sasaki et al^[87]^ found that low-dose linoleic acid inhibited peritoneal metastasis of gastric cancer cells through the activation of PPARγ and LOX-15 pathways. In a population study, Huang et al^[88]^ observed in a Chinese prospective cohort study that higher plasma levels of linoleic acid were associated with a reduced risk of gastric cancer and progression of precancerous lesions, particularly intestinal metaplasia. Furthermore, metabolic pathway enrichment analysis revealed that linoleic acid and its metabolites, such as α-linolenic acid and arachidonic acid, may participate in the occurrence of gastric cancer through lipid metabolism and inflammatory pathways. These studies suggest that the dual effects of linoleic acid may be related to its differential regulation of inflammatory pathways. At high doses, linoleic acid promotes the generation of inflammatory mediators through the COX-2/arachidonic acid pathway, thus supporting tumor growth and metastasis. However, at low doses, linoleic acid may inhibit tumor progression and promote apoptosis by activating anti-inflammatory pathways such as PPARγ. Therefore, the impact of linoleic acid on gastric cancer is complex, and its direction of action is regulated by dosage, metabolic pathways, experimental models, and individual metabolic backgrounds, warranting further exploration in multi-level mechanistic studies.

Butyrylcarnitine, a member of the acylcarnitine family, is responsible for transporting short-chain fatty acids (such as butyrate) into mitochondria for β-oxidation, generating acetyl-CoA, which then enters the tricarboxylic acid cycle to produce ATP.^[89]^ This process is significant in cancer cells. Studies have shown that butyrate, when used in combination with cisplatin, can increase apoptosis in gastric cancer cells through the mitochondrial apoptotic pathway. It is hypothesized that butyrylcarnitine may competitively inhibit mitochondrial fatty acid transport proteins, thereby affecting cancer cell energy metabolism, reducing oxidative stress, and inducing apoptosis. Research has demonstrated a negative correlation between butyrylcarnitine levels and the risk of various cancer types.^[90]^ Our study also found that butyrylcarnitine may act as a protective factor for gastric cancer, reducing the risk of its occurrence. Tsai et al^[91]^ found that butyrylcarnitine levels were significantly lower in gastric cancer tissues compared to adjacent healthy tissues. As a potential protective metabolite, butyrylcarnitine may play a role in the development of gastric cancer. Additionally, N-acetylglycine in our study also showed a negative correlation with gastric cancer risk, suggesting it may serve as a protective factor. N-acetylglycine is generated from glycine via N-acetyltransferase and is involved in cellular energy metabolism regulation as a product of amino acid metabolism. Studies have shown that N-acetylglycine participates in mitochondrial complex I-dependent reactions, thereby promoting the production of aspartate, which is crucial for cell survival in energy-deprived environments.^[92]^ Although no study has directly investigated the role of N-acetylglycine in gastric cancer, its involvement in cellular metabolism and energy balance suggests that it may play an auxiliary role in the development of gastric cancer. Future research should focus on metabolic pathway analysis, in vitro studies, and animal models to further validate the potential role of these metabolites in gastric cancer.

In addition to the aforementioned metabolites, our research has identified 3-methylhistidine, glutaroyl carnitine, X-12990 – docosapentaenoic acid (n6-DPA), and 3-(cystein-S-yl) acetaminophen as exhibiting a negative correlation with the risk of gastric cancer, suggesting their potential as protective factors against this disease. Conversely, 2-aminobutyrate, 1-stearoylglycerol (1-monostearin), and X-12095 – N1-methyl-3-pyridone-4-carboxamide – have shown a positive correlation with the risk of gastric cancer, indicating their inclusion as risk factors for this condition. Regrettably, the existing evidence regarding the causal association between these metabolites and gastric cancer remains largely theoretical. Currently, there is a scarcity of studies investigating these metabolites, necessitating further exploration of their relationship with gastric cancer.

Furthermore, it is noteworthy to acknowledge the need for investigation into the causal association between 7 unidentified metabolites (X-04357, X-11315, X-11334, X-12524, X-13859, X-11820, X-11540) and gastric cancer. Despite the lack of explicit documentation regarding these metabolites, our knowledge regarding them remains limited. Nevertheless, this limitation does not undermine the findings of our study. Conversely, the identification of these 7 unnamed blood metabolites in our research presents a novel avenue for future exploration in the realm of gastric cancer.

In summary, this study identified 21 blood metabolites with potential causal relationships to gastric cancer, including 12 metabolites negatively correlated and 9 positively correlated with disease risk. Notably, 3-methyl-2-oxovalerate, a branched-chain amino acid metabolic intermediate, exhibited protective effects through its negative association with gastric cancer risk. Serine demonstrated context-dependent roles: endogenous synthesis pathways suppressed tumor cell proliferation, while exogenous supplementation enhanced cisplatin sensitivity in resistant cases; however, high concentrations may promote tumor growth and metastasis. Piperine emerged as a protective factor, inhibiting cancer cell proliferation and suppressing Helicobacter pylori, a major gastric carcinogen. Butyrylcarnitine and N-acetylglycine also displayed protective associations. Linoleic acid exhibited dual effects, with high concentrations promoting tumor progression and low doses showing inhibitory activity. Metabolites such as X-11315 and others with limited supporting evidence require further investigation to clarify their roles in gastric cancer. These findings deepen our understanding of the metabolic landscape in gastric carcinogenesis and highlight potential biomarkers and therapeutic targets for future research.

Research has demonstrated that the heightened concentration of amino acids within the microenvironment constitutes a contributing factor to the process of carcinogenesis. Within our investigation, amino acid metabolites constituted 50% of the recognized metabolites and one-third of the overall metabolites. Specifically, 3-methyl-2-oxovalerate and 3-methylhistidine were implicated in 2 out of the 3 interconnected metabolite pathways. Consequently, amino acid metabolism emerges as a crucial connection in the initiation and progression of gastric cancer, thereby potentially serving as a diagnostic indicator and therapeutic target for this condition.

The current study encompassed a comprehensive spectrum of 486 human blood metabolites as potential exposure factors for MR Analysis, aiming to examine the metabolic characteristics of gastric cancer. Moreover, the use of MR methods effectively addresses the limitations associated with traditional observational studies and meta-analysis, such as confounding factors and reverse causality. While meta-analysis is powerful in synthesizing evidence across studies, it may introduce heterogeneity, which MR analysis helps to mitigate by using genetic variants as instruments to infer causal relationships.^[93,94]^ Additionally, the inclusion of Steiger directivity assessment and reverse MR Analysis in this investigation significantly mitigated the potential impact of reverse causality. Lastly, following stringent screening procedures, the obtained results exhibit a higher degree of scientific validity and methodological rigor. Nevertheless, it is important to acknowledge the limitations of this study. Firstly, due to the limited number of genome-wide significant SNPs identified for certain metabolites in existing GWAS, we had to relax the significance threshold from *P* <5 × 10^−8^ to *P* <5 × 10^−5^ during IVs selection to obtain sufficient IVs for analysis. Although this approach is a common strategy in MR studies, it may introduce weakly associated genetic variants, thereby increasing the risk of bias. To mitigate the impact of weak IVs, we calculated the *F*-statistics for all included SNPs, ensuring that all IVs had *F*-values >10. This statistical criterion for IVs strength enhances the robustness of causal inference to some extent. Secondly, all GWAS summary data used in this study were derived from individuals of European ancestry. While this selection reduces population stratification bias within the dataset, it may also limit the generalizability of our findings to non-European populations. Genetic architecture and metabolic profiles may vary significantly across races and ethnic groups, and these differences could influence metabolite expression, metabolic pathways, and gastric cancer risk. Therefore, caution is still warranted when extrapolating these results to other populations. Future studies should aim to incorporate multi-ethnic and multi-ancestry cohorts to validate these findings across diverse groups, thereby enhancing the universality of the conclusions. Additionally, exploring ethnicity-specific metabolic biomarkers may facilitate the discovery of novel methods for early gastric cancer screening and personalized therapeutic targets. Furthermore, while MR methods demonstrate clear advantages in eliminating confounding factors and clarifying causal directions, the specific biological mechanisms underlying several metabolites identified in this study remain poorly understood in the context of gastric cancer development. Some metabolites lack substantial foundational or epidemiological evidence, and conflicting effect directions have even been reported in prior studies. Such bidirectional regulatory characteristics may arise from multifactorial influences, including dosage, tissue specificity, and tumor stage. Consequently, future investigations should integrate in vitro and in vivo experiments with larger-scale clinical studies to comprehensively elucidate the functional roles of these metabolites in gastric cancer metabolic reprogramming, signaling pathway modulation, and drug response processes, thereby validating and expanding our findings. Additionally, in our MR analysis, we identified potential causal associations between certain blood metabolites and gastric cancer risk. However, we acknowledge that gastric cancer progression itself may induce systemic metabolic changes. Previous studies have shown that as gastric cancer advances, patients exhibit significant metabolic disturbances, including abnormalities in amino acid metabolism, lipid metabolism, and energy pathways.^[8]^ These changes may affect the levels of various blood metabolites and potentially confound observed associations. Since the genetic instruments used in MR analyses are determined prior to disease onset, this approach can effectively reduce the risk of reverse causality bias. Nonetheless, we recognize the limitations of this method. Future research should integrate longitudinal metabolomic data to monitor changes in metabolite levels across different stages of gastric cancer progression, thereby providing a more comprehensive understanding of the interplay between metabolic alterations and disease development.

## 5. Conclusions

In summary, this study investigated the causal relationship between blood metabolites and gastric cancer using MR analysis with genome-wide data. Our findings revealed potential causal associations between 21 blood metabolites and gastric cancer, identifying 3 metabolic pathways potentially involved in gastric carcinogenesis. Notably, 3-methyl-2-oxovalerate represents a promising therapeutic target warranting further investigation. The roles of serine and linoleic acid as either protective or risk factors for gastric cancer require additional research to reconcile currently inconsistent findings. Clinically, 2-hydroxyisobutyrate and butyrylcarnitine may serve as potential diagnostic biomarkers for gastric cancer. The antitumor effects of piperine have been validated in most studies, yet further clinical trials are needed to confirm its safety and efficacy. These findings provide valuable directions for early screening, prevention, and treatment of gastric cancer, as well as the design of future clinical studies.

## Acknowledgments

The authors sincerely thank all participants and investigators for the contributions of GWAS data.

## Author contributions

**Conceptualization:** Sulan Chen.

**Data curation:** Bin Zhang, Song Wang, Ming Yang.

**Formal analysis:** Sulan Chen.

**Investigation:** Qiaohui Shen, Rui Zhang.

**Visualization:** Sulan Chen, Bin Zhang.

**Writing – original draft:** Sulan Chen.

**Writing – review & editing:** Sulan Chen, Yan Leng.

## Supplementary Material


