# Supplementary material for: Blood metabolites and gastric cancer risk: A bidirectional 2-sample Mendelian randomization study
Source: Medicine (Baltimore). 2025 Jul 18;104(29):e43339. doi: 10.1097/MD.0000000000043339 (PMC12282805; doi:10.1097/MD.0000000000043339)
Supplement: Supplementary file 2 [file medi-104-e43339-s002.docx]

**Supplementary Figure**

Supplementary Figure 1. Scatter plot of MR analysis results between 21 blood metabolites.

| 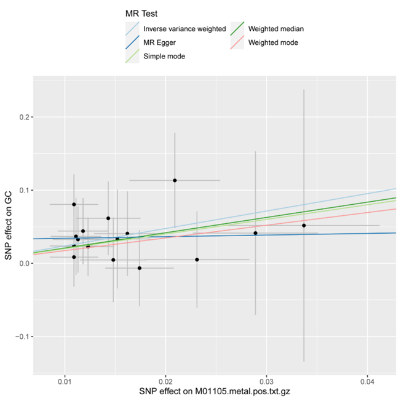 | 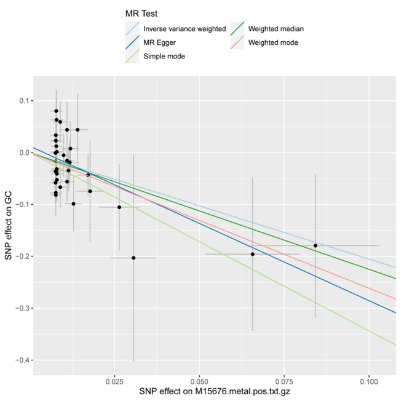 | 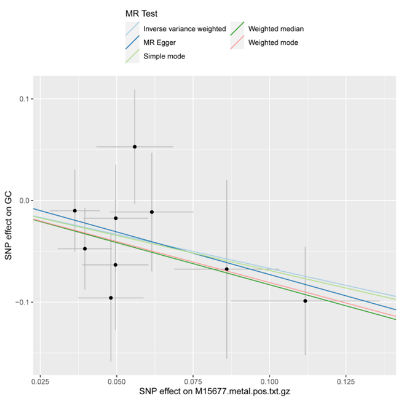 |
| --- | --- | --- |
| A: linoleate (18:2n6) | B: 3-methyl-2-oxovalerate | C: 3-methylhistidine |
| 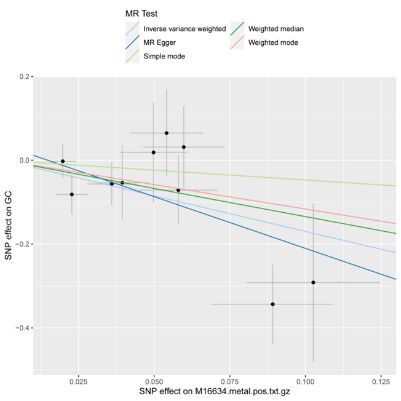 | 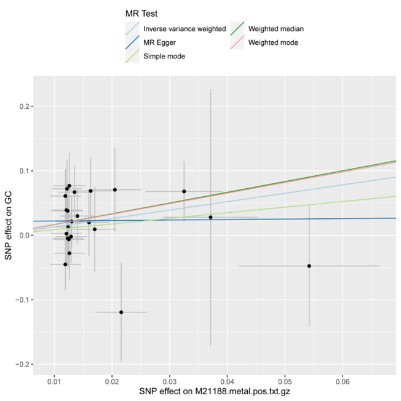 | 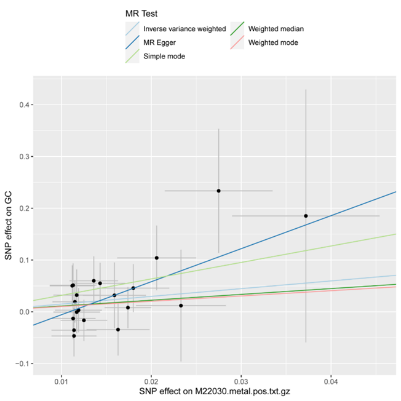 |
| D: X-04357 | E: 1-stearoylglycerol (1-monostearin) | F: 2-hydroxyisobutyrate |
| 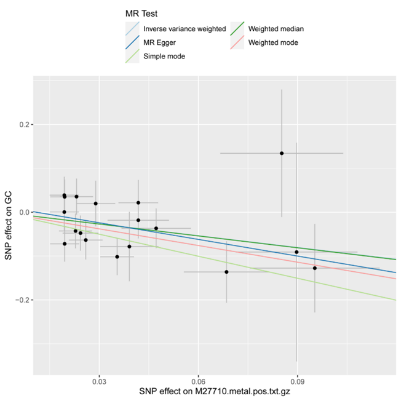 | 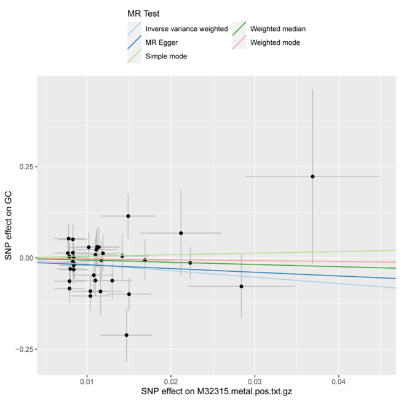 | 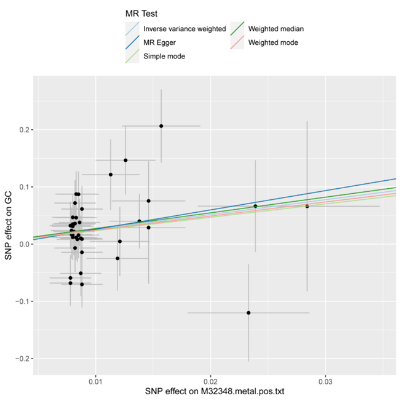 |
| G: N-acetylglycine | H: serine | I: 2-aminobutyrate |
| 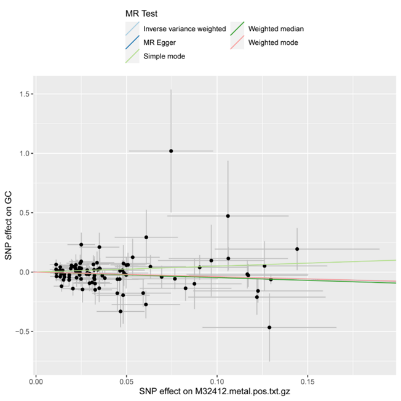 | 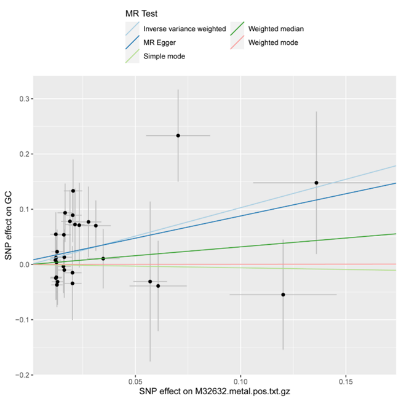 | 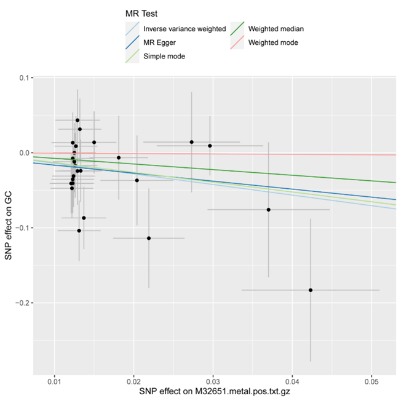 |
| G: butyrylcarnitine | K: X-11315 | L: X-11334 |
| ;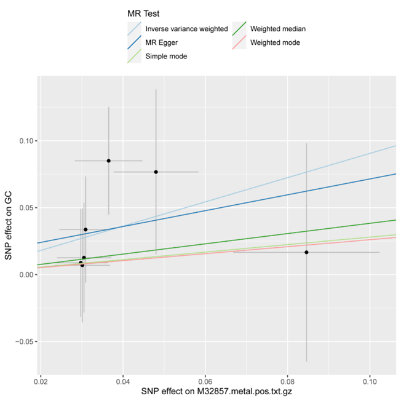 | 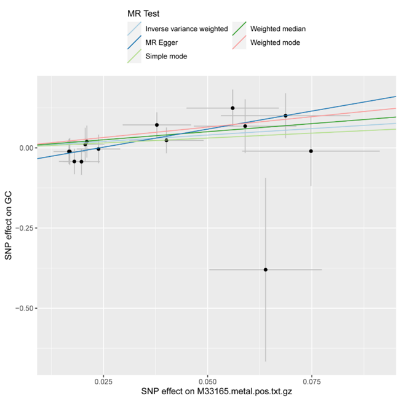 | 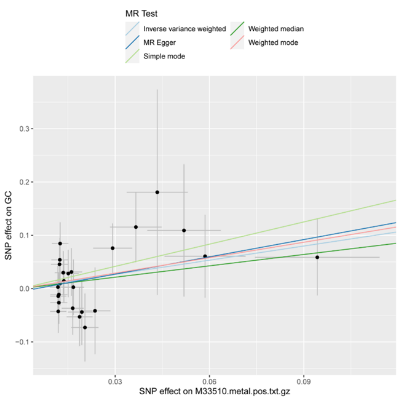 |
| M: X-11540 | N: X-11820 | O: X-12095--N1-methyl-3-pyridone-4-carboxamide |
| 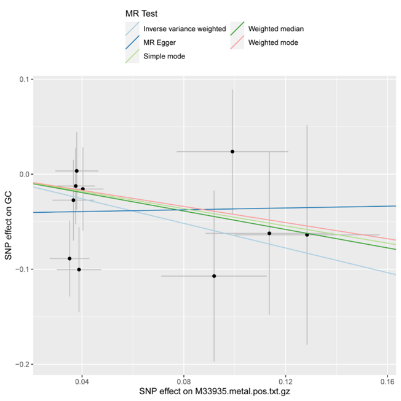 | 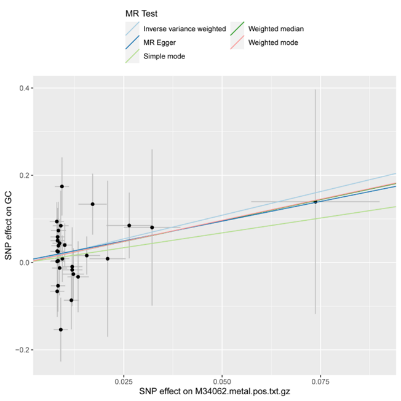 | 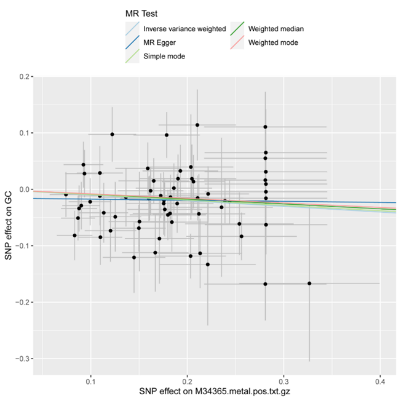 |
| P: piperine | Q: X-12524 | R: 3-(cystein-S-yl)acetaminophen* |
| 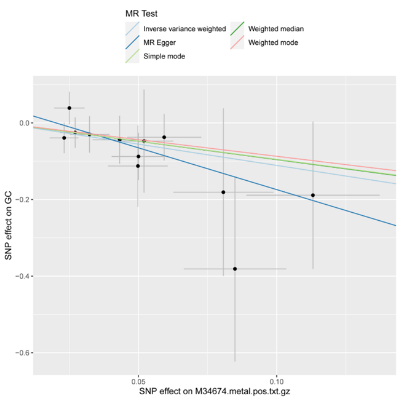 | 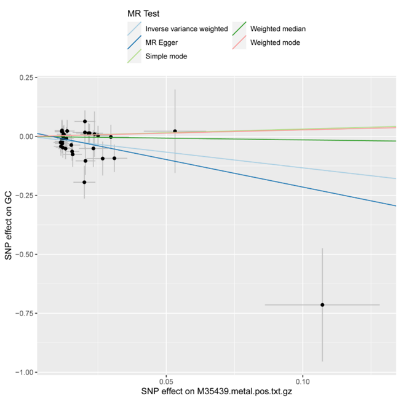 | 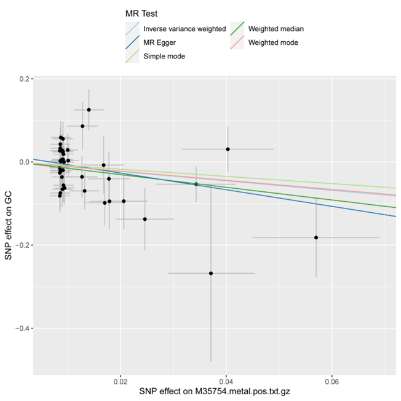 |
| S: X-12990--docosapentaenoic acid (n6-DPA) | T: glutaroyl carnitine | U: X-13859 |

Supplementary Figure 2. Leave-one-out plot of MR analysis results between 21 blood metabolites.

| 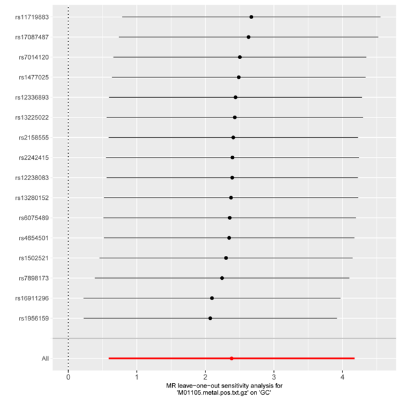 | 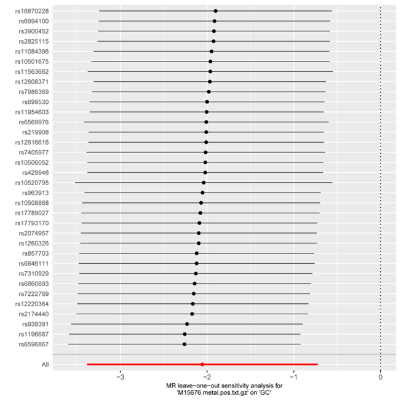 | 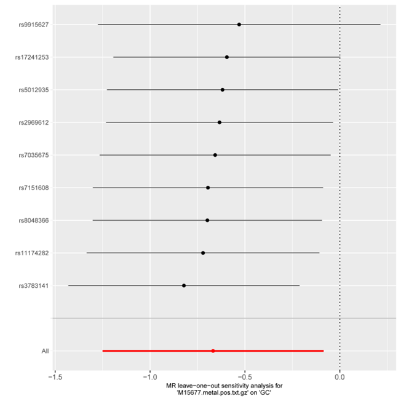 |
| --- | --- | --- |
| A: linoleate (18:2n6) | B: 3-methyl-2-oxovalerate | C: 3-methylhistidine |
| 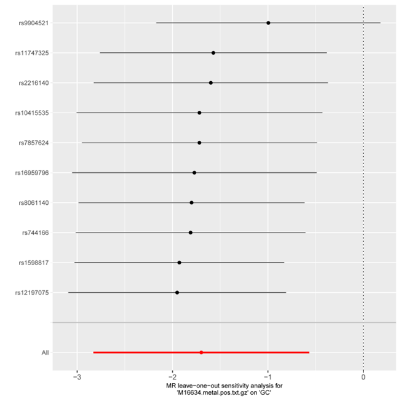 | 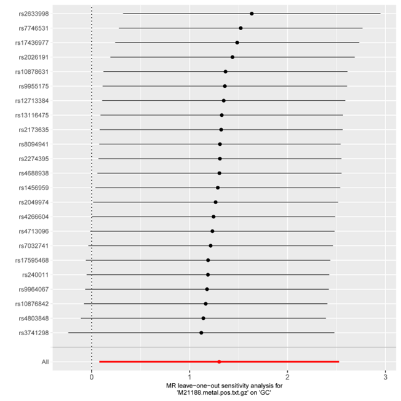 | 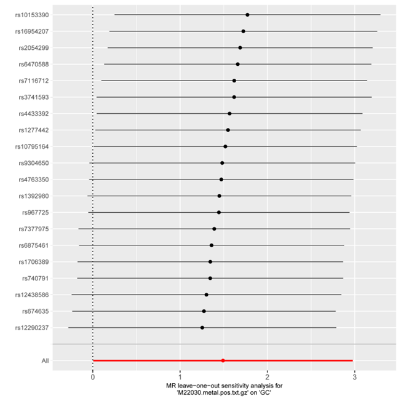 |
| D: X-04357 | E: 1-stearoylglycerol (1-monostearin) | F: 2-hydroxyisobutyrate |
| 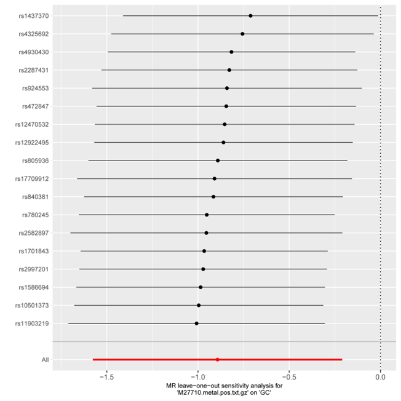 | 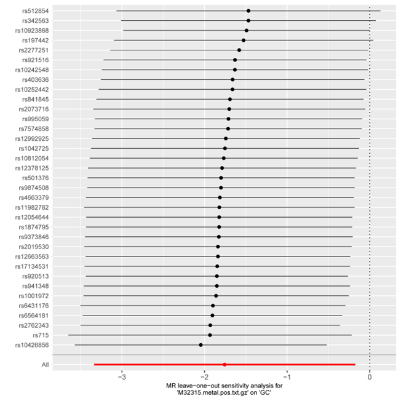 | 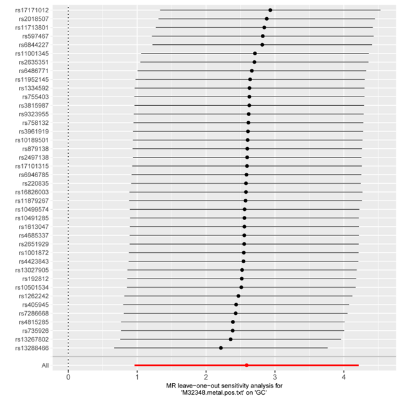 |
| G: N-acetylglycine | H: serine | I: 2-aminobutyrate |
| 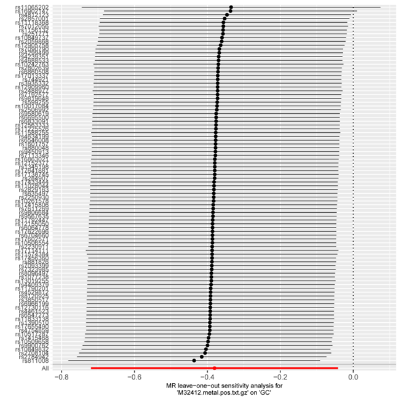 | 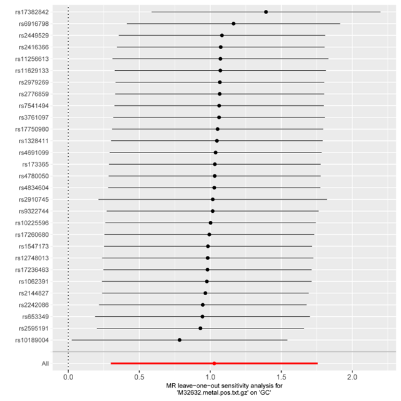 | 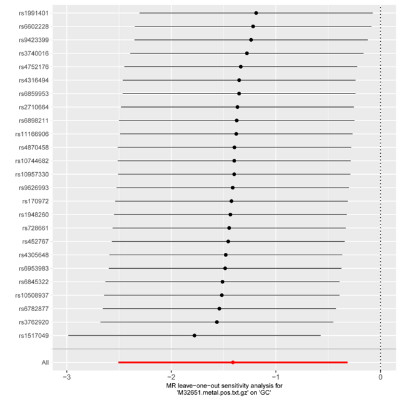 |
| G: butyrylcarnitine | K: X-11315 | L: X-11334 |
| 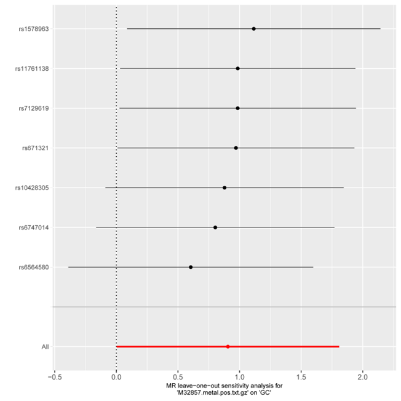 | 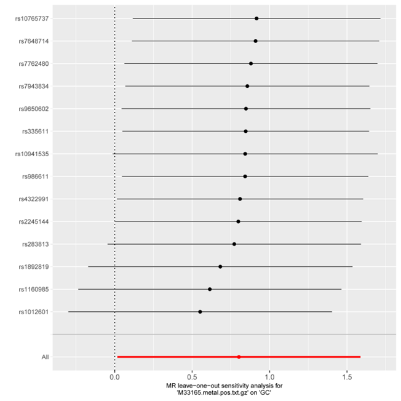 | 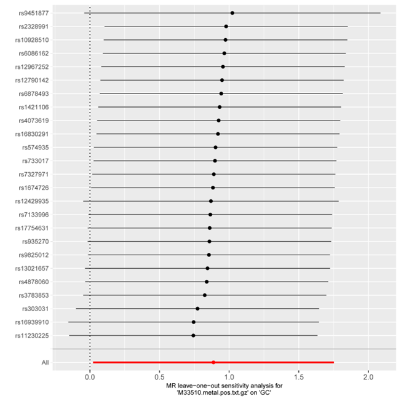 |
| M: X-11540 | N: X-11820 | O: X-12095--N1-methyl-3-pyridone-4carboxamide |
| 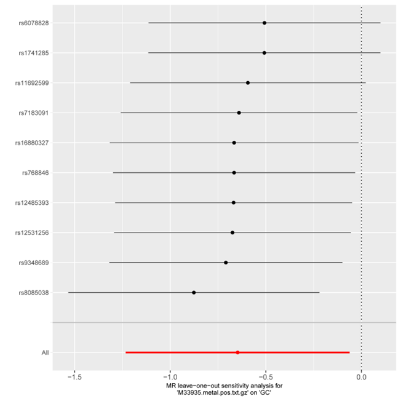 | 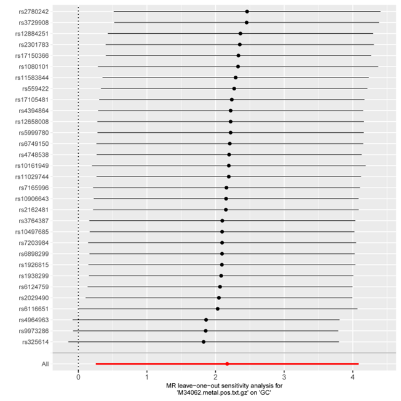 | 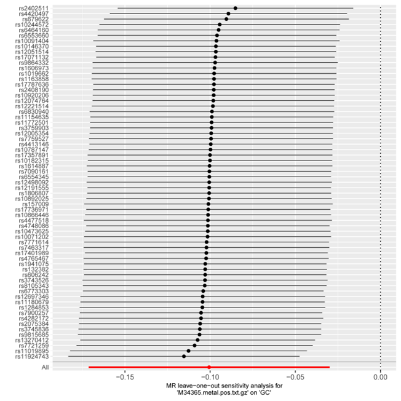 |
| P: piperine | Q: X-12524 | R: 3-(cystein-S-yl)acetaminophen* |
| 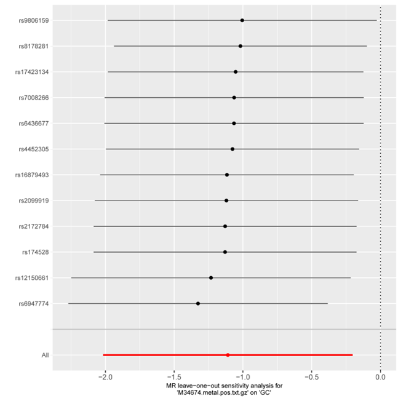 | 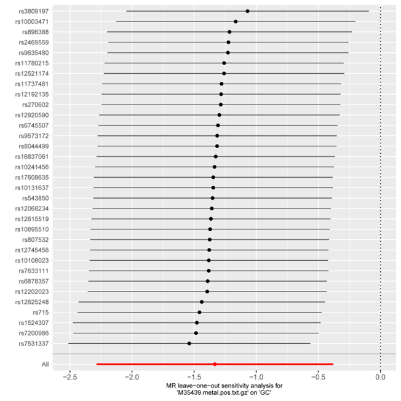 | 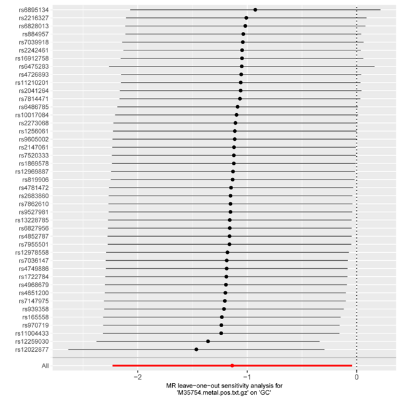 |
| S: X-12990--docosapentaenoic acid (n6-DPA) | T: glutaroyl carnitine | U: X-13859 |
